# Revisiting Atopy: The IgE-Dependent Amplification Loop as a Forgotten Driver of Atopic Dermatitis

**DOI:** 10.3390/pathophysiology33020041

**Published:** 2026-06-22

**Authors:** Ryoji Tanei, Yasuko Hasegawa

**Affiliations:** 1 Department of Dermatology, Tokyo Metropolitan Institute for Geriatrics and Gerontology, Itabashi, Tokyo 173-0015, Japan; 2 Department of Geriatric Pathology, Tokyo Metropolitan Institute for Geriatrics and Gerontology, Itabashi, Tokyo 173-0015, Japan

**Keywords:** atopy revisited, epicutaneous allergen exposure, extrinsic atopic dermatitis, FcεRI-expressing antigen-presenting cells, house dust mite (HDM) allergens, IgE-dependent amplification loop, IgE-mediated delayed-type hypersensitivity, inflammatory dendritic epidermal cells, Langerhans cells, spongiosis

## Abstract

Atopic dermatitis (AD) is increasingly interpreted through frameworks emphasizing barrier dysfunction, type 2 cytokine signaling, pruritus pathways, and microbial dysbiosis, often relegating IgE-mediated mechanisms to secondary roles. In this narrative review, we synthesize historical, clinical, immunologic, and histopathologic evidence to propose a conceptual model in which IgE-bearing antigen-presenting cells (APCs)—including Langerhans cells, inflammatory dermal dendritic cells, and inflammatory dendritic epidermal cells (IDECs)—participate in an IgE-dependent amplification loop that may contribute to the chronicity of extrinsic (IgE-associated) AD. Evidence from human studies indicates that FcεRI-expressing APCs can acquire environmental allergens through IgE, enhancing antigen uptake and T-cell activation, while mast cells and basophils further reinforce type 2 inflammation through IgE-dependent and IgE-augmented pathways. Although these mechanisms have been described across distinct experimental and clinical contexts, their integration into a unified pathogenic circuit remains hypothesis-driven. We therefore present an interpretive framework that organizes these partially validated mechanisms into a coherent model linking cutaneous sensitization, allergen capture, APC activation, Th2 polarization, and spongiosis formation. This conceptual synthesis aims to reposition IgE-mediated processes within the broader pathophysiology of extrinsic AD and to highlight potential therapeutic implications for targeting IgE–FcεRI signaling and IgE-dependent APC biology.

## 1. Introduction

Atopic dermatitis (AD) has long been recognized as a chronic, relapsing, pruritic inflammatory skin disease characterized by eczematous lesions, complex etiology, and heterogeneous endotypes [1,2]. Historically, however, its classical definition—rooted in the early work of Sulzberger and colleagues [3]—placed immunoglobulin (Ig)E-mediated atopy at the center of its pathophysiology [4,5,6]. Over the past two decades, this view has shifted toward a barrier-centric and interleukin (IL)-4/IL-13-dominated paradigm, driven largely by the discovery of filaggrin mutations [7] and the clinical success of type 2 cytokine blockade [8]. At the same time, the therapeutic efficacy of the anti-IgE monoclonal antibody omalizumab has proven limited, even in extrinsic (IgE-associated) AD [9], reinforcing the perception that IgE plays only a secondary or epiphenomenal role in disease pathogenesis [2,10].

This contemporary view stands in contrast with two well-established observations. First, the atopic march—a concept widely accepted in Western allergy research—consistently identifies cutaneous IgE sensitization as the initiating event linking infantile AD to food allergy, allergic rhinitis, and asthma [11]. Second, extrinsic AD, which accounts for the majority of AD cases in both Western and Asian populations (~80% [1,12]), is characterized by markedly elevated serum IgE levels that correlate strongly with disease severity [12,13]. Together, these findings suggest that IgE may contribute more directly to AD pathogenesis than is currently acknowledged.

These discrepancies, which highlight the involvement of distinct pathological processes in AD, may be reconciled by a mechanistic framework that integrates past research with recent advances in immunology [14]. Within this framework, the pathology in patients with a high-IgE extrinsic AD phenotype is characterized by the following features: In non-lesional skin, FcεRI-expressing Langerhans cells (LCs) that bind IgE may silently capture environmental allergens (e.g., dust mites, pollens, food-derived proteins), thereby establishing a persistent state of cutaneous sensitization. In lesional skin, exposure to these allergens induces IgE-loaded antigen-presenting cells (APCs)—including LCs, inflammatory dendritic epidermal cells (IDECs), and inflammatory dermal dendritic cells (DCs)—to act as potent amplifiers of type 2 inflammation via an IgE–FcεRI-dependent pathway, primarily through enhanced Th2 activation. This amplified type 2 inflammatory milieu provides a strong activating environment for mast cells, eosinophils, and basophils. In addition, these IgE-loaded APCs promote the infiltration of T lymphocytes, and together these cell populations contribute to the formation of eczematous lesions (spongiotic dermatitis) in AD [14,15]. Taken collectively, these mechanisms may result in an IgE-driven amplification loop that acts as a potent booster of the type 2 inflammation induced by impaired barrier conditions and upstream cytokine signaling, thereby further escalating the inflammatory milieu [5,14,16]. Although each of these processes is supported by experimental or clinical evidence, they have not yet been demonstrated as a single, fully integrated pathogenic pathway.

This model may provide a unifying explanation for several unresolved features of AD, including the strong association between high IgE levels and severe extrinsic AD, the link between AD and subsequent allergic diseases, the limited therapeutic efficacy of anti-IgE monoclonal antibodies, and the incomplete response to IL-4/IL-13 blockade in some patients. Importantly, it reframes IgE not as a downstream marker of type 2 inflammation but as an active driver of disease in the IgE-dependent endotype of AD.

This review synthesizes emerging evidence to propose an IgE-dependent amplification loop as a central pathogenic mechanism in a distinct endotype of AD and discusses its implications for disease classification and therapeutic response.

## 2. Rethinking Atopy: From Classical IgE-Mediated Allergy to a Multilayered Immune Concept

Atopy was originally conceptualized in the early to mid-20th century based on clinical observations that eczema, asthma, and allergic rhinitis arise from a shared immunological predisposition involving hypersensitivity reactions mediated by “reagins” [17], later identified as IgE [18]. Within this classical framework, the mechanisms by which IgE—typically associated with immediate-type hypersensitivity—might contribute to the development of eczematous lesions in AD, traditionally attributed to delayed-type hypersensitivity (DTH), remained poorly understood. Nevertheless, clinical data consistently demonstrated strong associations between elevated serum total IgE levels, the presence of allergen-specific IgEs, and the extrinsic phenotype of AD. As a result, IgE was incorporated into global diagnostic criteria for AD [19] and came to be regarded as a major pathogenic mediator. Importantly, these associations do not in themselves establish causality, but they provide a foundation for interpreting IgE as a potential contributor to disease mechanisms.

Subsequent advances in skin immunology, however, have revealed that IgE participates in a broader spectrum of immune responses than previously appreciated. Beyond its canonical role in immediate hypersensitivity (15–30 min), IgE can activate eosinophils via mast cells to induce late-phase responses (6–12 h) [20], enhance T-cell-mediated delayed-type responses (24–72 h) by amplifying DC function through FcεRI binding [4,5], and contribute to spongiosis formation through antigen presentation in the epidermis [14,15]. IgE also signals through CD23 (FcεRII), a low-affinity receptor expressed mainly on B cells, where it regulates antigen transport and IgE production [16]. Collectively, these findings indicate that atopy is not a single pathway defined solely by IgE-mediated mast-cell activation but instead represents a multilayered immune state.

Despite this historical context, the importance of IgE in AD is currently being underestimated. Over the past two decades, the discovery of filaggrin loss-of-function mutations [7] and the clinical success of IL-4/IL-13 inhibitors [8] have reinforced a paradigm in which barrier dysfunction and type-2 cytokines are viewed as the primary drivers of AD. In parallel, the limited therapeutic efficacy of anti-IgE biologics in extrinsic AD [9] has further diminished the perceived contribution of IgE-mediated pathways. Advances in the understanding of itch mechanisms [21,22,23], innate immunity [10,24], and *Staphylococcus aureus*-driven skin dysbiosis [25] have consolidated this view, suggesting that AD is driven predominantly by barrier impairment, immune dysregulation, pruritus, and microbial imbalance [26,27]. Consequently, elevated IgE levels in AD have come to be regarded merely as downstream markers of inflammation rather than mechanistic contributors.

However, this interpretation is difficult to reconcile with several key observations. Extrinsic AD—characterized by marked elevations in serum total and allergen-specific IgE levels that correlate strongly with disease severity—represents the predominant phenotype in both Western and Asian populations [1,12,13,28]. Moreover, longitudinal studies of atopic disease consistently demonstrate that cutaneous IgE sensitization is the earliest immunological event linking infantile AD to subsequent food allergy, allergic rhinitis, and asthma [11]. Taken together, these findings suggest that IgE may play a more direct role in AD pathophysiology than is reflected in current models emphasizing barrier dysfunction, innate immune activation, itch–scratch cycles, allergen/antigen exposure, *S. aureus*-driven dysbiosis, and the type-2 inflammation collectively driven by these processes.

Meanwhile, mast cells and basophils—traditionally viewed as central effector cells in IgE-mediated immediate hypersensitivity (mast cells in the skin, basophils in the blood)—are now recognized to contribute to both innate and adaptive immune responses in AD. During adaptive immune responses, these cells are activated by IgE-dependent stimuli and release a broad array of mediators, including IL-4, IL-13, TNF-α, tryptase, and histamine [28,29]. This activation also induces the production of chemotactic cytokines (alarmins) such as thymic stromal lymphopoietin (TSLP), IL-25, and IL-33 [14,30], as well as chemokines including CCL17, CCL22, and CXCL10. These mediators—released predominantly from keratinocytes and inflammation-recruited myeloid cells such as macrophages and DCs—collectively create a highly inflammatory microenvironment [28,29,30]. IgE-mediated late-phase responses, driven primarily by mast cells and eosinophils but also involving basophils, neutrophils, and Th2 cells, further sustain the recruitment of innate and adaptive immune cells after allergen exposure [6,20,31]. Furthermore, in IgE-mediated DTH, DCs expressing IgE via FcεRI can induce T-cell-mediated inflammation—sometimes accompanied by infiltration of group 2 innate lymphoid cells (ILC2s) and natural killer (NK) cells—independently of mast-cell degranulation, suggesting a mechanistic link between innate and adaptive immunity [32]. These findings indicate that IgE functions not only as an initiator of immediate allergic responses but also as a broader amplifier of cutaneous immunity.

Taken together, these historical, clinical, and immunologic observations indicate that the traditional view of atopy as a purely IgE-mediated immediate hypersensitivity state is overly narrow. IgE participates in a multilayered network of innate and adaptive immune pathways that extends far beyond mast-cell and basophil degranulation, influencing antigen uptake, DC activation, mast-cell- and eosinophil-driven inflammation, and T-cell-mediated responses. The strong association between elevated IgE levels and the extrinsic AD phenotype, along with the central role of cutaneous IgE sensitization in the atopic march, further suggests that IgE contributes directly to disease pathogenesis rather than serving merely as a downstream marker of type-2 inflammation. Importantly, this interpretation synthesizes findings from distinct experimental contexts and is intended as a conceptual framework rather than a claim that all mechanisms operate as a single empirically validated sequence. These considerations provide the rationale for re-examining the role of IgE in AD and set the stage for a closer look at FcεRI-expressing DC populations that may mediate IgE-dependent amplification of cutaneous inflammation, as well as their interplay with FcεRI-bearing effector cells such as mast cells and basophils.

The following sections provide an overview of the pathophysiological research on AD, with a focus on the mechanisms underlying IgE-mediated extrinsic AD driven by house dust mite (HDM), the most representative environmental allergen in this disease. HDM-driven pathology is broadly comparable to that induced by other inhaled allergens, including pollens [5,14].

## 3. IgE-Dependent Cutaneous Immune Network: FcεRI-Expressing Dendritic Cells, Mast Cells, and Basophils

### 3.1. Langerhans Cells: Silent Antigen Collectors and Central Players in AD Pathogenesis

LCs reside in the steady-state epidermis as tissue-resident DCs defined by their CD1a^+^, CD207^+^, and HLA-DR^+^ phenotype and by expression of the high-affinity IgE receptor FcεRI. In AD, LCs function as a pivotal immunologic hub with both pro- and anti-inflammatory capacities, integrating signals from barrier disruption, type-2-skewed immunity, microbial interactions such as *S. aureus*, and chronic inflammatory circuits that shape disease progression [33]. In both lesional and non-lesional skin of patients with extrinsic AD, LCs bind circulating IgE via FcεRI and can capture environmental protein antigens penetrating the epidermis—including aeroallergens, microbial antigens, and food-derived proteins. This phenomenon becomes detectable when serum total IgE levels exceed approximately 300 IU/mL and is consistently observed at levels above 3000–4000 IU/mL [34,35]. Notably, FcεRI expression on LCs correlates strongly with serum total IgE concentrations. Moreover, FcεRI expressed on LCs and other DC subsets differs structurally from the αβγ_2_ tetrameric complex found on mast cells and basophils, instead existing as an αγ_2_ trimer lacking the β chain required for signal amplification [16,36].

**Figure 1:**
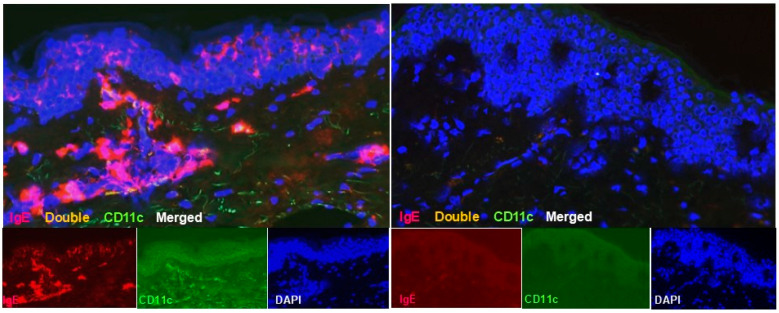
Immunohistopathological comparison of uninvolved skin in extrinsic (IgE-associated) AD and normal skin in allergic rhinitis. Uninvolved skin of extrinsic AD (**left**): Regular arrangements of IgE-expressing LCs (red) are observed in the epidermis. These LCs exhibit markedly elongated IgE-bearing dendrites, some of which extend beyond tight junctions into the area beneath the stratum corneum, suggesting readiness to capture allergens penetrating from the skin surface. Numerous IgE-expressing cells (red) are also present in the dermis, many of which are presumed to be mast cells. Only a very small number of IgE^+^CD11c^+^ inflammatory dermal DCs (yellow) are additionally detectable. Normal skin of a patient with allergic rhinitis (**right**): Epidermal LCs do not express IgE, and scarcely any IgE-expressing cells are present in the dermis. Original magnification: 200×. In double immunofluorescence staining, nuclei are labeled with 4′,6-diamidino-2-phenylindole (DAPI; blue). Images reproduced, with partial modifications, with permission from the authors and journal (*International Journal of Molecular Sciences*) [14]. Characteristics of the patients shown: Left: 61-year-old woman, whose disease began at age 15, with a serum total IgE level of 10,198 IU/mL and high HDM-specific IgE titers [14,15]. Right: 50-year-old man with a normal serum total IgE level of 46 IU/mL and low HDM-specific IgE titers. Abbreviations: AD, atopic dermatitis; CD, cluster of differentiation; DC, dendritic cell; HDM, house dust mite; IgE, immunoglobulin E; LC, Langerhans cell.

In the non-lesional skin of patients with extrinsic AD and markedly elevated serum IgE levels, LCs expressing IgE are arranged in regular patterns within the epidermis (Figure 1, left). These LCs exhibit elongated IgE-bearing dendrites, some extending beyond tight junctions into the region beneath the stratum corneum, appearing poised to capture allergens entering from the skin surface [14]. Such features are absent in healthy controls and in individuals with atopic diseases who possess allergen-specific IgEs but maintain normal total IgE levels (Figure 1, right).

Although direct histopathologic detection of allergens such as HDMs—the major environmental allergens in AD—within IgE-expressing LCs has been reported only infrequently [14,37], it is highly plausible that allergen sampling occurs in non-lesional skin. This assumption is supported by evidence of reduced barrier function in non-lesional skin of extrinsic AD patients [38]. Moreover, previous studies have reported a case in clinically normal skin of individuals with an atopic constitution who had already become sensitized after one year of allergen exposure, in whom HDM antigens penetrated the skin through hair follicles and subsequently induced contact urticaria [39]. Atopy patch tests further demonstrate that higher doses of HDM antigens yield higher rates of positive reactions, suggesting that low-dose allergen exposure alone may not induce overt inflammation even when captured by LCs [40]. Although directly visualized evidence of this process remains limited, these converging observations provide reasonable inferential support for silent allergen capture by LCs in non-lesional skin.

After capturing allergens, these LCs migrate to draining lymph nodes, where they enhance Th2-cell differentiation and promote the production of allergen-specific IgEs through cytokine-mediated stimulation—primarily IL-4, with contributions from IL-13 [27,36]. Such IgE-dependent allergen sampling may occur without overt inflammation, establishing a state of persistent cutaneous sensitization [33]. Environmental exposure data support this concept: patients with extrinsic AD often live in settings with higher HDM burdens than healthy individuals [41], and major HDM antigens (Der p 1 and Der f 1) are detectable on the skin surface of 93% of healthy volunteers [42]. This ubiquitous exposure provides a plausible basis for the establishment of persistent cutaneous sensitization in extrinsic AD.

**Figure 2:**
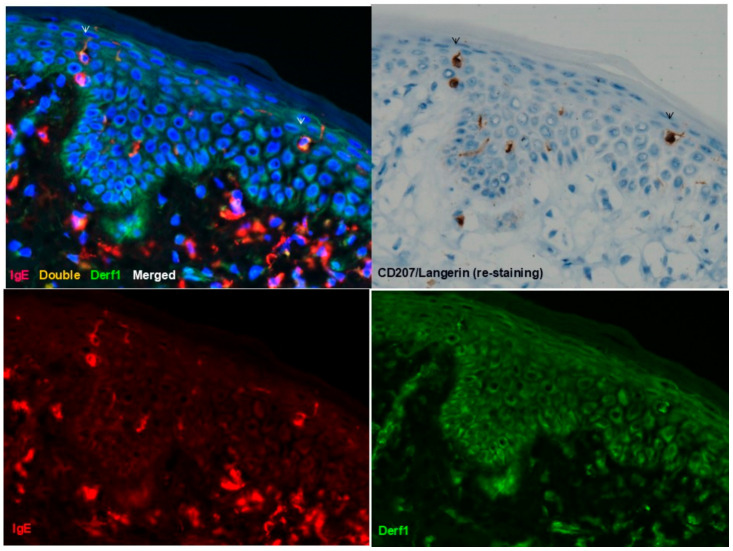
Immunohistopathology of lichenified eczema in extrinsic (IgE-associated) AD. LCs capturing HDM allergens via IgE in the non-spongiotic epidermis: Double-positive IgE^+^ Der f1^+^ cells (yellow) with elongated dendrites extending into the tight-junction region are present beneath the tight junction (arrowheads). Immunohistochemical re-staining indicates that these double-positive IgE^+^ Der f1^+^ cells in the epidermis are positive for CD207 (arrowheads), identifying them as LCs. Lymphocytic infiltration is scarcely observed around these LCs. Original magnification: 200×. In double immunofluorescence staining, nuclei were labeled with 4′,6-diamidino-2-phenylindole (DAPI), although the single-channel DAPI image is not included in the figure. Images reproduced with permission from the authors and journal (*Dermatology Clinics & Research*) [43]. Characteristics of the patient shown: 84-year-old man, whose disease began at age 50, with a serum total IgE level of 19,757 IU/mL and high HDM-specific IgE titers [43]. Abbreviations: AD, atopic dermatitis; CD, cluster of differentiation; Der f, *Dermatophagoides farinae*; HDM, house dust mite; IgE, immunoglobulin E; LC, Langerhans cell.

In chronic lesional skin of extrinsic AD patients with sufficiently high total and HDM-specific IgE levels, IgE-expressing LCs capturing HDM antigens are observed histopathologically within non-spongiotic areas—often in the perifollicular epidermis, where the stratum corneum barrier is relatively weak, and occasionally in the interfollicular epidermis [14,15]. Some of these LCs extend dendritic branches beyond tight junctions to capture HDM antigens (Figure 2) [43]. However, few lymphocytes are found in direct contact with IgE-expressing, HDM-bearing LCs within the epidermis. Histopathologic analyses indicate that approximately 60% of extrinsic AD patients with serum HDM-specific IgEs exhibit detectable HDM antigens in chronic lesional skin, at significantly higher levels than patients with non–HDM-sensitized AD [37].

In lesional AD skin, LCs (CD1a^+^) expressing the costimulatory molecules CD86 and CD80—canonical providers of the second signal required for T-cell activation—are increased in number [44]. Despite this increase, previous reports have shown that the total number of epidermal LCs (CD1a^+^) is reduced in AD lesional skin compared with non-lesional AD skin and healthy controls [45]. Together with the observation that HDM-capturing LCs rarely form contacts with lymphocytes within the chronic, non-spongiotic epidermis, these findings suggest that LCs are unlikely to engage in local antigen presentation to T cells within the epidermis. Instead, they are thought to migrate from the epidermis into the dermis and subsequently to the draining lymph nodes, where they participate in antigen presentation and are believed to produce and secrete Th2-inducing cytokines and chemokines either during migration or upon arrival in the lymph nodes. Through this antigen-presenting function, LCs promote type-2-skewed immune responses, including Th2-cell activation and the induction of allergen-specific IgE [33,36]. Recent single-cell RNA sequencing (scRNA-seq) analyses further show that LCs under inflammatory conditions in AD upregulate CCL17, increase expression of FcεRI (*FCER1A*), and exhibit enhanced transcriptional signatures associated with T-cell activation, enhancing their capacity to shape a Th2-polarized microenvironment [46]. In LCs that have captured HDM antigens via IgE, IgE has been demonstrated to play a crucial role in facilitating antigen presentation to T cells [35]. Importantly, these elements derive from studies conducted in distinct experimental and clinical contexts, and their integration here is intended as a conceptual synthesis rather than a claim that all mechanisms have been demonstrated within a single unified system.

### 3.2. Inflammatory Dermal Dendritic Cells: Key Drivers of Th2-Skewing in the Dermis

In AD lesions, inflammatory monocyte-derived myeloid DCs infiltrate the skin and differentiate into two major subsets based on their localization: IDECs in the epidermis and inflammatory dermal DCs in the dermis [47,48,49]. Both subsets express high levels of FcεRI and carry greater IgE loads than LCs in the lesional skin of high-IgE extrinsic AD patients [32,43,50]. Because inflammatory dermal DCs share many phenotypic markers with IDECs (e.g., CD11c^+^, FcεRI^+^, HLA-DR^+^, CD207^−^), these two inflammatory DC populations are thought to exhibit functional plasticity [43,47,51]. Although they can be broadly distinguished by CD1a expression—CD1a^+^ for IDECs and CD1a^−^ for inflammatory dermal DCs—this marker is not fixed and may shift depending on the inflammatory milieu [43,47].

**Figure 3:**
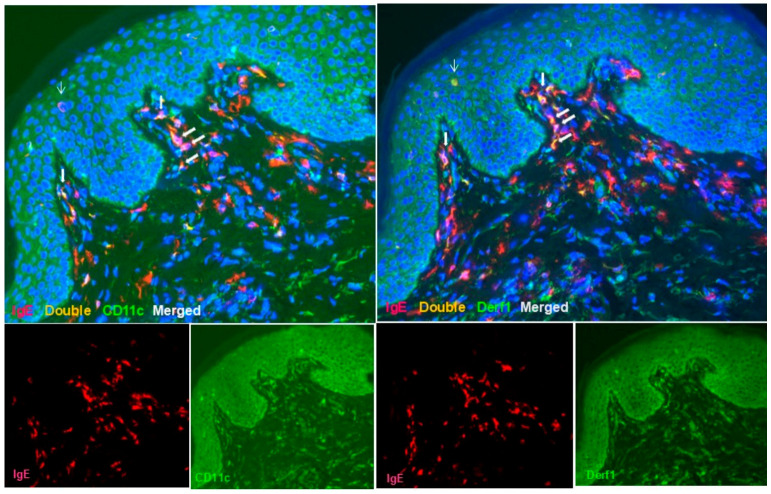
Immunohistopathology of lichenified eczema in extrinsic (IgE-associated) AD. IgE-expressing inflammatory dermal DCs capturing HDM allergens in the upper dermis: Double-positive IgE^+^ CD11c^+^ cells (yellow; **left**) and double-positive IgE^+^ Der f1^+^ cells (yellow; **right**) are observed in the upper dermis of the AD lesion. In these sections, the majority of IgE^+^ CD11c^+^ cells and IgE^+^ Der f1^+^ cells in the papillary dermis show similar morphology and localization (arrows). These double-positive cells are in contact with small round cells, suggesting that inflammatory dermal DCs capturing HDM allergens may present antigen to lymphocytes in this area. In contrast, co-localization of an IgE^+^ CD11c^−^ cell—representing an HDM-capturing LC—and an IgE^+^ Der f1^+^ cell is also observed in the epidermis (arrowheads), without accompanying small round-cell infiltration. Original magnification: 200×. Sets of figures represent serial sections. In double immunofluorescence staining, nuclei were labeled with 4′,6-diamidino-2-phenylindole (DAPI), although the single-channel DAPI image is not included in the figure. Images reproduced with permission from the authors and journal (*Dermatology Clinics & Research*) [43]. Characteristics of the patient shown: Same patient as in Figure 2. Abbreviations: AD, atopic dermatitis; CD, cluster of differentiation; DC, dendritic cell; Der f, *Dermatophagoides farinae*; HDM, house dust mite; IgE, immunoglobulin E; LC, Langerhans cell.

Inflammatory dermal DCs efficiently capture environmental allergens such as HDM antigens via FcεRI-bound IgE and present them to T cells (Figure 3) [15,16,43]. They also promote the activation and proliferation of OX40-expressing Th2 cells through expression of the costimulatory molecule OX40 ligand (OX40L), a feature particularly induced under keratinocyte-derived TSLP stimulation. Through this pathway, they contribute to the production and release of type-2 inflammatory mediators—including IL-4, IL-13, IL-5, and IL-31—from Th2 cells, and chemokines such as CCL17, CCL18, and CCL22 from the DCs themselves [13,47,52,53]. This type-2 immune activation further impairs keratinocyte barrier function, thereby perpetuating and amplifying the disease process [2]. A particularly important aspect of this pathomechanism is that IgE–FcεRI-facilitated antigen uptake enhances antigen presentation efficiency by approximately 100- to 1000-fold compared with IgE-independent pathways, enabling DCs to activate T cells even under extremely low allergen exposure conditions [16,54,55]. These estimates derive from well-established experimental systems and are widely used as reference values in the IgE–FcεRI literature, although they do not necessarily reflect a single defined physiological setting.

In addition to inflammatory dermal DCs, several other DC subsets contribute to shaping the immune landscape in AD. Conventional type 2 DCs (cDC2), particularly when activated by keratinocyte-derived TSLP, express high levels of OX40L and, through antigen presentation together with OX40L-mediated co-stimulation, act as key instructors of T-cell differentiation, efficiently driving Th2 polarization and expansion and reinforcing type-2-dominant inflammation within the dermis [53,56]. Plasmacytoid DCs, specialized antiviral sentinels capable of producing large amounts of type I interferons, also express FcεRI and can bind IgE, suggesting that they may participate in IgE-associated immune modulation in AD [16,47,57]. Another distinct population, the 6-sulfo LacNAc-expressing monocytes (formerly referred to as slanDCs), functions as a potent amplifier of inflammation by rapidly releasing high levels of pro-inflammatory cytokines—most notably TNF-α and IL-1β—allowing them to act as powerful ignition cells within inflamed skin [58].

Collectively, these DC populations—together with other immune and stromal cells such as monocytes, macrophages, T cells, B cells, mast cells, eosinophils, basophils, fibroblasts, peripheral sensory neurons, and keratinocytes—form a highly interconnected inflammatory circuit within the AD dermis and its draining lymph nodes. Through extensive bidirectional crosstalk, these cells sustain and amplify type-2 inflammation, ultimately shaping the chronic and relapsing nature of AD. The mechanisms highlighted here represent those most relevant to IgE-dependent pathways in high-IgE extrinsic AD, while also intersecting with broader immunologic processes that contribute to AD pathophysiology more generally.

### 3.3. Inflammatory Dendritic Epidermal Cells: IgE-Dependent Amplifiers Linking Delayed-Type Hypersensitivity to Spongiosis in Atopic Dermatitis

IDECs represent a hallmark population of inflammatory DCs that accumulate within the epidermis of lesional AD. First characterized by Wollenberg, Bieber, and colleagues, IDECs are defined by their CD1a^+^, CD11b^+^, CD11c^+^, CD206^+^, CD207^−^, and FcεRI^+^ phenotype and by their emergence under inflammatory rather than steady-state conditions [59,60]. Like inflammatory dermal DCs, a defining feature of IDECs is their ability to capture environmental allergens via FcεRI-bound IgE, enabling highly efficient antigen uptake even when allergen concentrations are extremely low. IgE-facilitated antigen internalization can enhance antigen presentation efficiency by nearly three orders of magnitude compared with IgE-independent pathways [16,54,55]. In extrinsic AD, where serum IgE levels are markedly elevated, this mechanism becomes a central amplifier of epidermal inflammation.

Notably, like LCs, inflammatory dermal DCs, and the cDC2 subset described earlier, IDECs also express the costimulatory molecules CD80 and CD86; however, their expression of these molecules is far more pronounced than that of LCs, endowing IDECs with substantially greater T-cell-stimulatory capacity [59]. What further distinguishes IDECs is their epidermal localization and their uniquely high expression of FcεRI, which together enable efficient IgE-dependent allergen capture and position IDECs as specialized amplifiers of DTH within the epidermis [50,60].

Through a series of immunohistopathological studies conducted in middle-aged and older adult patients with AD, our group has demonstrated that IDECs play a crucial role in the development of eczematous lesions—specifically the histopathologically defined spongiotic dermatitis—arising from IgE-mediated DTH responses in extrinsic AD with HDM sensitization. These studies include:

(1) Evidence of IgE-positive epidermal DCs bearing HDM antigens—i.e., LCs and IDECs—in naturally occurring AD lesions, with histopathologic features closely resembling those observed in HDM-induced positive atopy patch test reactions;
(2) A conceptual paper integrating these findings with prior literature to propose the IgE-mediated DTH model of spongiosis formation in extrinsic AD;
(3) A comparative study demonstrating disease-specific patterns of spongiosis across AD and other skin disorders, reinforcing the central role of IDECs in AD-associated spongiotic dermatitis.

Together, these investigations identify IDECs as the central effector cells driving spongiosis formation in extrinsic AD [14,15,32,43]. While these studies provide a coherent interpretive framework, we recognize that they derive largely from our own morphological and immunohistopathological analyses, and thus should be viewed within the context of the current limitations of IDEC-focused research.

**Figure 4:**
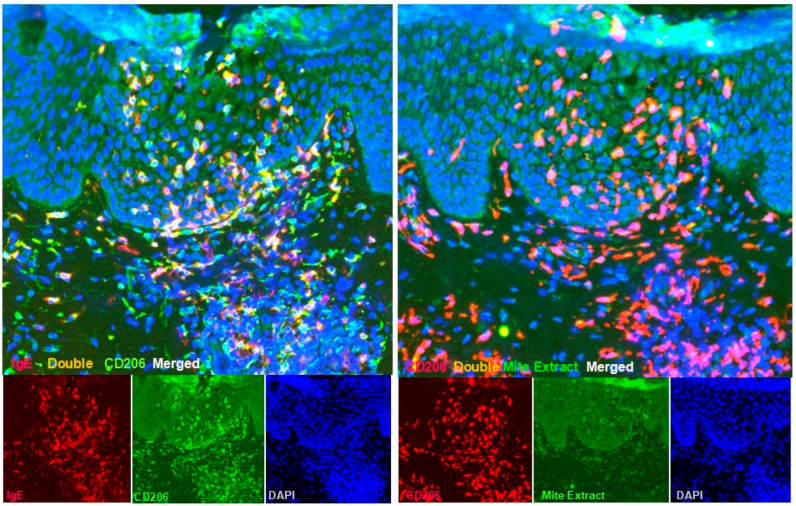
Immunohistopathology of lichenified eczema in extrinsic (IgE-associated) AD. Accumulation of IgE-expressing IDECs in the spongiotic epidermis: IgE^+^ CD206^+^ IDECs infiltrate and aggregate within focal spongiosis, and most of these IDECs capture HDM (mite extract) antigens (yellow in the epidermis of the left and right sections). CD4^+^ T cells also infiltrate this spongiotic epidermis, suggesting that IgE-mediated DTH is occurring in this area [14]. Original magnification: 200×. Sets of figures represent serial sections. In double immunofluorescence staining, nuclei are labeled with 4′,6-diamidino-2-phenylindole (DAPI; blue). Images reproduced with permission from the authors and journal (*Dermatopathology*) [15]. Characteristics of the patient shown: Same AD patient as described in Figure 1. Abbreviations: AD, atopic dermatitis; CD, cluster of differentiation; DC, dendritic cell; DTH, delayed-type hypersensitivity; HDM, house dust mite; IDEC, inflammatory dendritic epidermal cell; IgE, immunoglobulin E; LC, Langerhans cell.

In these morphological studies—conducted both in naturally occurring AD lesions presenting with lichenified eczema and in HDM-induced positive atopy patch test reactions—IDECs, which were only sparsely scattered within the subepidermal layer of non-spongiotic epidermis in naturally occurring lesions and essentially absent in uninvolved skin at patch-test sites, became markedly accumulated within spongiotic epidermis. Within these regions, IDECs colocalized with HDM antigens, a finding observed in approximately 60% of naturally occurring AD cases, as demonstrated by double immunofluorescence staining (Figure 4) [14,15]. CD4^+^ T cells infiltrated these areas in close proximity, positioning them to receive antigen presentation from IgE-expressing IDECs.

In addition to IDECs, HDM-capturing LCs—which exhibit weaker IgE expression—also infiltrate spongiotic areas. However, as spongiosis progresses and IDEC aggregates become prominent, these LCs tend to disperse toward the periphery of the spongiotic regions. With further progression, when spongiotic vesicles become apparent, aggregated IDECs show little residual colocalization with HDM antigens, and ultimately form dense clusters that are expelled from the epidermis [43]. This sequence of changes is thought to reflect the time-course kinetics of spongiosis formation [14,32]. These kinetic inferences are based on serial morphological patterns rather than direct longitudinal tracking, and should therefore be interpreted as a plausible model consistent with available evidence.

This kinetic pattern may indicate a functional transition in IDECs—consistent with their role as inflammatory DCs [49]—from an early phase dominated by antigen presentation and the induction of inflammation, to a later phase characterized by antigen sequestration and progressive elimination of captured antigens, and finally to a phase that promotes extrusion of foreign material toward the epidermal surface.

Building on these morphological and temporal observations, converging findings from in vitro, ex vivo, and in vivo studies collectively reveal a pathomechanistic cascade in which IDECs emerge as key IgE-dependent modulators linking DTH responses to the formation of spongiosis in extrinsic AD. As spongiosis begins to develop, activated LCs within the epidermis upregulate chemokines such as CCL2 (MCP-1) [50], creating a microenvironment that preferentially recruits monocyte-derived IDECs from the dermis toward emerging spongiotic foci [14,15,43].

Once accumulated within spongiotic epidermis, IDECs capture HDM allergens through high-affinity IgE–FcεRI-mediated uptake and process them into MHC class II-bound peptides [5], which they then present to allergen-specific Th1 cells, triggering the release of IFN-γ and TNF-α [14,50]. In parallel with antigen presentation, IDECs secrete cytokines including IL-1α, IL-12, IL-16, and IL-18, as well as chemokines such as CCL2 and CCL3 (MIP-1α), in response to allergen stimulation [5,36,50,54], thereby enhancing the recruitment and activation of multiple T-cell subsets—including Th1, Th2, and Th22 cells [61]—within the inflamed tissue. Notably, however, our immunohistochemical analyses did not detect IL-12 expression in IDECs accumulated within spongiotic foci [32], suggesting that IL-12 production by IDECs may be context-dependent or less prominent in vivo.

Keratinocytes within spongiotic regions further contribute to this inflammatory circuit by producing TSLP, CCL22, and CXCL10, which attract Th2 and Th1 cells and reinfmorce a local microenvironment that sustains antigen presentation and T-cell engagement [14,62]. Together, cytokines produced by IDECs or induced through IDEC-mediated T-cell activation—such as IFN-γ, TNF-α, IL-1α, and IL-18—along with these keratinocyte-derived mediators, generate inflammatory signals that activate keratinocytes and, in turn, induce intercellular edema, disrupt tight junctions, and drive both the initiation and progression of spongiosis.

Within this cytokine-driven epithelial response, IDECs function as central integrators of both IFN-γ-driven spongiotic formation and type-2 cytokine-mediated amplification, thereby coordinating the evolution of spongiotic pathology and sustaining the IgE-dependent inflammatory loop that defines extrinsic AD. Importantly, this cascade is based on a DTH reaction uniquely amplified by IgE–FcεRI interactions, distinguishing extrinsic AD from other eczematous disorders [14,32,63].

Recent scRNA-seq studies have identified *FCER1A*^+^ myeloid clusters enriched in lesional AD skin; however, these datasets do not explicitly present the costimulatory, chemokine, or pattern-recognition receptor features characteristic of IDECs [64]. Therefore, scRNA-seq data currently provide limited insight into the classical IDEC population central to atopy, indicating that morphological and immunophenotypic analyses remain essential for defining IgE-dependent pathways in AD.

Together, the morphological, immunological, and transcriptomic data converge to position IDECs as IgE-dependent epidermal amplifiers that integrate allergen capture, antigen presentation, T-cell activation, and keratinocyte dysfunction to drive the spongiotic pathology characteristic of extrinsic AD. Notably, this synthesis reflects the current state of evidence, acknowledging that IDEC-centered models remain constrained by the methodological limitations inherent to human tissue studies and the absence of direct in vivo kinetic tracking.

### 3.4. Mast Cells and Basophils: IgE-Dependent Innate–Adaptive Amplifiers

In extrinsic AD, mast cells expressing IgE are markedly increased within the upper dermis of chronically inflamed lesions [28,51], underscoring their central involvement in disease pathophysiology. Classically regarded as the principal effector cells of IgE-mediated immediate-type hypersensitivity and functioning as tissue-resident sentinels, mast cells orchestrate both the immediate response and the early phase of the IgE-dependent late-phase response in AD [31]. Upon allergen-induced crosslinking of surface-bound IgE, mast cells release histamine, tryptase, TNF-α, IL-4, IL-5, and IL-13, which increase vascular permeability and promote the initial influx of leukocytes. They also generate lipid mediators such as prostaglandin D_2_ and leukotrienes, as well as chemokines including CCL5 (RANTES), CCL3, and CCL2, which strongly drive the recruitment of eosinophils, neutrophils, basophils, DCs, and T cells into the skin [28,65]. The accumulation of these mediators amplifies type-2-driven chronic inflammation and contributes clinically to the persistence of erythema with inflammatory infiltrates and to ongoing pruritus. Furthermore, this inflammatory milieu promotes sustained eosinophil infiltration, and eosinophil-derived mediators in turn enhance mast-cell activation, establishing a self-perpetuating cycle of inflammation [28]. These pathways reflect the well-recognized overlap between IgE-dependent activation and broader innate–adaptive inflammatory circuits, which cannot be cleanly separated in the context of chronic AD.

Basophils represent another IgE-responsive effector population that participates in innate–adaptive amplification, particularly in acute allergic skin inflammation. They accumulate in IgE-mediated late-phase responses [31] and in atopy patch test lesions [66], where they produce large amounts of IL-4 and thereby enhance local Th2 inflammation [27,29]. However, in AD lesions, basophils are detected in only approximately 60% of cases and their infiltration levels are generally modest [67], likely because basophils are blood-resident cells that predominantly respond during acute-phase inflammation—making basophil-rich acute lesions difficult to capture in a disease that is fundamentally chronic and relapsing. Nevertheless, when allergens reach the skin via the bloodstream—such as in food-related allergy [68] or inhalant-related systemic exposure exemplified by HDM-associated “oral mite anaphylaxis” [69]—basophils may play roles equal to or even exceeding those of mast cells. Patients with extrinsic AD, particularly infants and young children (in whom food sensitization is common) and a subset of adults, are often sensitized not only to aeroallergens such as HDM but also to a wide range of dietary allergens [28,70]. Consequently, hematogenous allergen exposure may serve as an important exacerbating factor contributing to skin inflammation and pruritus [6].

However, because AD patients typically exhibit heightened regulatory T-cell (Treg) activity in both lesional and non-lesional skin as well as in peripheral blood [52,71], and because gastrointestinal absorption of allergens varies substantially depending on digestive conditions and commonly used medications [72], skin reactions triggered by hematogenous allergen exposure are more likely to manifest as exudative inflammatory erythema rather than simple urticarial wheals [14,73]. Although basophils have been proposed as key orchestrators in murine models of IgE-dependent chronic allergic inflammation (IgE-CAI) [29]—a process distinct from human IgE-mediated DTH because mouse DCs lack FcεRI and do not express IgE [74]—an analogous mechanism has not yet been clearly demonstrated in AD patients. Even so, in scenarios involving hematogenous allergen exposure, basophil-driven responses resembling IgE-CAI in mice may possibly occur in humans [75].

Importantly, mast cells and basophils function as potent sources of IL-4, a cytokine that amplifies IgE-dependent immune pathways by promoting Th2 differentiation, driving the production of allergen-specific IgEs, and upregulating FcεRI expression on LCs, IDECs, and inflammatory dermal DCs [27,28,29]. Through this upstream IL-4 signaling, both cell types enhance the capacity of FcεRI-expressing DCs to capture IgE-bound allergens and drive allergen-specific T-cell responses in extrinsic AD. Mast cells further shape cutaneous immunity through direct crosstalk with keratinocytes: mast-cell-derived IL-4 and IL-13 induce keratinocytes to produce CCL22, thereby recruiting CCR4^+^ Th2 cells into lesional sites [28,50]. Mast cells also stimulate keratinocytes to produce TSLP, in part through mast-cell-derived tryptase acting via protease-activated receptor-2 (PAR-2) on keratinocytes [76]. Moreover, IL-13 derived from mast cells and Th2 cells induces dermal fibroblasts to produce periostin—a matricellular protein that reinforces type-2 inflammation by acting on keratinocytes—which in turn further enhances TSLP production, establishing a TSLP–periostin amplification loop that drives the chronicity of Th2-skewed inflammation [77]. In this context, these interconnected pathways are synthesized here to illustrate how IgE-responsive effector cells interface with the wider inflammatory network, rather than to imply that all downstream events are exclusively IgE-dependent.

Collectively, mast cells and basophils act as IgE-dependent innate–adaptive amplifiers that operate in parallel with IgE-loaded, FcεRI-expressing DCs. While IDECs activate both type-2 and type-1 inflammatory pathways and serve as key epidermal drivers linking DTH to spongiosis, mast cells and basophils provide powerful upstream and peripheral signals that intensify type-2 inflammation, augment IgE–FcεRI-mediated antigen uptake, and sustain the chronic inflammatory loop characteristic of extrinsic AD. These mechanisms are presented as components of the IgE-dependent axis most relevant to high-IgE extrinsic AD, while acknowledging that they intersect with broader inflammatory pathways that contribute to AD pathophysiology in general.

## 4. The IgE-Dependent Amplification Loop: An Integrated Pathomechanistic Model

As outlined in the previous section, FcεRI-expressing DCs, mast cells, and basophils form a multilayered IgE-dependent inflammatory network that operates across the epidermis, dermis, and draining lymph nodes—a network that becomes progressively more consolidated as IgE sensitization intensifies in extrinsic AD. LCs silently capture environmental allergens and establish persistent cutaneous sensitization while regulating the initiation and resolution of inflammation; inflammatory dermal DCs drive Th2 polarization within the dermis; IDECs amplify DTH responses and orchestrate spongiosis formation in the epidermis; and mast cells and basophils provide potent innate–adaptive signals that intensify type-2 inflammation and enhance FcεRI-mediated antigen uptake.

Rather than functioning as isolated pathways, these IgE-dependent mechanisms converge to create a self-reinforcing inflammatory circuit that is unique to extrinsic AD. In this section, we build on the functional framework of IgE–FcεRI-expressing cells described above to delineate the architecture of the IgE-dependent amplification loop and clarify its role within the broader pathophysiology of AD. Notably, this loop is presented as an integrative conceptual model derived from converging but partial lines of evidence, rather than as a fully validated experimental circuit.

### 4.1. Architecture of the IgE-Dependent Amplification Loop

**Figure 5:**
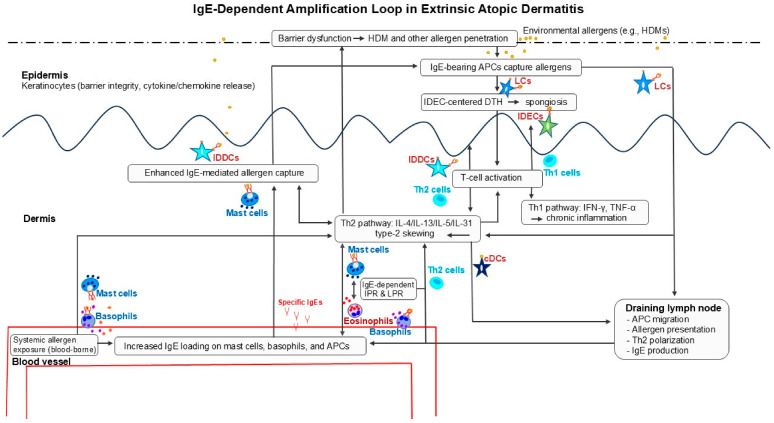
IgE-dependent amplification loop in extrinsic (IgE-associated) AD. This figure illustrates the spatially organized IgE-dependent amplification loop, integrating barrier dysfunction, IgE–FcεRI-mediated allergen capture, antigen presentation, T-cell activation, keratinocyte responses, and cytokine-driven feedback that collectively sustain chronic inflammation in extrinsic AD. Abbreviations: AD, atopic dermatitis; APC, antigen-presenting cell; CD, cluster of differentiation; cDC2, conventional type 2 dendritic cell; DTH, delayed-type hypersensitivity; HDM, house dust mite; IDDC, inflammatory dermal dendritic cell; IDEC, inflammatory dendritic epidermal cell; IFN, interferon; IgE, immunoglobulin E; IL, interleukin; IPR, immediate phase response; LC, Langerhans cell; LPR, late phase response; Th, T helper.

The IgE-dependent amplification loop in extrinsic AD is best understood as a spatially organized, multicellular circuit that integrates barrier dysfunction, allergen handling, antigen presentation, and cytokine-driven feedback into a single self-reinforcing system. Rather than operating as a linear cascade, the loop consists of interconnected modules (functional units) distributed across the epidermis, dermis, and draining lymphoid tissues, each contributing distinct but complementary roles that collectively sustain chronic inflammation (Figure 5). Because this circuit functions as a dynamic loop rather than a unidirectional pathway, the modules are not intended to represent a strict temporal or hierarchical sequence but instead reflect interacting components of a continuously reinforcing system.

At the entry point of the loop, impaired barrier structures—including weakened tight junctions and altered stratum corneum integrity—permit low-dose environmental allergens to access the viable epidermis. Once inside, allergens are rapidly intercepted by FcεRI-bearing cells loaded with IgE and positioned at different anatomical layers. LCs, IDECs, inflammatory dermal DCs, mast cells, and basophils each participate in allergen capture, but their contributions differ according to their tissue localization and activation thresholds. This distributed allergen-sampling system ensures that even minimal allergen exposure is efficiently detected and incorporated into the circuit.

Within the epidermis, LCs—residing quietly as antigen-sampling sentinels—function as a hub that orchestrates the initiation, amplification, and resolution of cutaneous inflammation. In contrast, IDECs act as the principal amplifiers of local immune activation. Their residence in the lower epidermal layers—perhaps a strategic placement for impending barrier leakage—together with their high FcεRI expression and robust cytokine/chemokine output, enables them to coordinate the recruitment and retention of T cells. Keratinocytes, which themselves serve as major sources of immune-danger signals, respond to cytokines released by activated lymphocytes and DCs and further destabilize the barrier through tight-junction disruption and intercellular edema. As increasing amounts of allergens penetrate the epidermis, keratinocytes mount a tissue-level defensive reaction aimed at expelling these foreign substances, manifested morphologically as spongiosis [14]. However, in AD, this spongiotic response paradoxically exacerbates barrier instability, facilitates additional allergen entry, and thereby strengthens the initiation module of the IgE-dependent amplification loop.

In the dermis, inflammatory dermal DCs integrate signals from IgE-bound allergens with high efficiency. Upon exposure to epithelial-derived cytokines such as TSLP, they upregulate OX40L and acquire an enhanced capacity to prime allergen-specific Th2 cells, thereby linking cutaneous barrier dysfunction to adaptive type-2 immunity. Mast cells and basophils, through their rapid release of IL-4, IL-13, and other mediators, provide an essential bridge between innate and adaptive immunity. Their cytokine output not only enhances FcεRI expression on DC subsets but also helps sustain a type-2-skewing cytokine milieu that promotes IgE availability, in part through crosstalk with keratinocytes, eosinophils, fibroblasts, and Th2 cells. This, in turn, strengthens the loop that contributes to the chronicity of extrinsic AD.

Beyond the skin, systemic allergen exposure further reinforces this circuit. Circulating allergens—originating from respiratory or dietary sources, including HDM antigens—engage IgE-loaded basophils in the peripheral blood, triggering IL-4 release [78] and upregulating FcεRI on skin-homing DCs. In parallel, these same allergens can reach the dermis, where they are intercepted by mast cells positioned as the first sentinels, before integrating into the dermal and epidermal modules of the loop and thereby linking systemic allergen exposure with cutaneous amplification mechanisms.

These epidermal and dermal modules converge through lymphatic trafficking of antigen-bearing DCs, which deliver processed peptides to T cells in the draining lymph nodes. The resulting expansion of Th2-skewed lymphocytes and the promotion of allergen-specific IgE production feed back into the skin, where Th2-derived cytokines—including IL-4, IL-13, IL-5, and IL-31—perpetuate keratinocyte dysfunction, chemokine production, chronic pruritus, and FcεRI upregulation, while IgE further amplifies this loop through enhanced FcεRI engagement on cutaneous immune cells. Through this cyclical reinforcement, the loop becomes self-sustaining, capable of maintaining inflammation even in the absence of overt allergen surges. Together, these spatially and functionally integrated components form the architectural basis of the IgE-dependent amplification loop. In the context, this framework is intended to delineate the IgE-dependent axis most relevant to high-IgE extrinsic AD, while acknowledging that it intersects with broader inflammatory pathways shared across AD.

The construction of this IgE-dependent amplification loop provides a mechanistic basis for the strong correlations observed in highly IgE-sensitized individuals between disease severity, total serum IgE levels, and the breadth of allergen-specific IgE repertoires. As the loop becomes more deeply engaged, the cutaneous manifestations diversify: depending on the route and intensity of allergen exposure, patients may present with eczematous dermatitis, infiltrated erythema, or exudative inflammatory flares. In extrinsic AD—where polysensitization to environmental and food-derived allergens is common—these cutaneous manifestations frequently coexist. Moreover, as IgE sensitization progresses, autoreactivity toward keratinocyte-derived self-antigens can emerge [79], enabling activation of the loop even in the absence of exogenous allergen exposure, such as through scratching alone. Consequently, once the IgE-dependent amplification loop is engaged, extrinsic AD tends to follow a chronic and treatment-refractory course.

### 4.2. Integrating the IgE-Dependent Amplification Loop into the Broader Pathophysiology of AD

This section integrates the IgE-dependent amplification loop with established non-IgE-mediated pathways in AD, highlighting how the proposed model fits within the broader and multifactorial pathophysiology of the disease. Current concepts frame AD as a heterogeneous inflammatory skin disease shaped by genetic predisposition and environmental exposures, in which barrier dysfunction, innate immune activation, pruritus–scratch cycles, allergen/antigen penetration, *Staphylococcus aureus*-driven dysbiosis, and the resulting type-2-skewed inflammation collectively construct and maintain the disease state. At the same time, AD—like asthma and allergic rhinitis—is fundamentally a hypersensitivity disorder. Within this broader framework, the IgE-dependent amplification loop can be viewed as a hypersensitivity-boosting module that becomes particularly prominent in extrinsic AD.

Across the natural history of AD, patients typically progress from a subclinical phase to a non-IgE phase, followed by an IgE-sensitized phase that defines extrinsic AD, with some individuals eventually developing autoallergic AD characterized by IgE reactivity to keratinocyte-derived self-antigens, whereas others maintain an intrinsic AD phenotype throughout their disease course [80]. Although individual trajectories are shaped by genetic and environmental factors, these patterns represent a continuous spectrum rather than discrete categories [1]. Importantly, evidence from repeated-elicitation contact hypersensitivity models in mice [81] and from human mosquito-bite hypersensitivity reactions [82] indicates that DTH-type responses often precede IgE-dependent hypersensitivity. This suggests that the “non-IgE phase” of AD may already contain latent, environmentally driven DTH responses rather than reflecting purely innate hyper-responsiveness. This layered hypersensitivity architecture also helps explain why non-IgE-mediated mechanisms—chiefly DTH-type pathways—continue to contribute to disease activity even in extrinsic AD [83], and why anti-IgE therapy such as omalizumab shows only partial efficacy [9].

Clinical observations further support this view. In indeterminate AD [14], where serum total IgE levels are normal but allergen-specific IgEs are detectable, LCs and DCs do not express IgE, indicating that the IgE-dependent amplification loop is not fully assembled; yet IgE-mediated immediate and late-phase responses can still occur. Interestingly, in extrinsic AD patients whose LCs do not express IgE—despite detectable allergen-specific IgE, a pattern typically seen in individuals with only modest elevations in total serum IgE and resembling the indeterminate phenotype—epicutaneous exposure to HDM-dominant house dust allergen extract via atopy patch testing can still elicit eczematous reactions, occurring in 40% of tested cases. In contrast, among extrinsic AD patients who possess IgE-expressing LCs—representing IgE-mediated DTH—the positivity rate rises to 90% [35]. Mouse models further demonstrate that, after allergen-specific IgE sensitization, epicutaneous allergen challenge can induce spongiosis following the late-phase response [84]. Together, these findings indicate that spongiosis formation in extrinsic AD under epicutaneous allergen exposure can arise not only through IgE-mediated DTH but also through (i) IgE-driven late-phase responses and downstream reactions within this pathway [84,85], and (ii) potentially coexisting IgE-independent DTH mechanisms [83]. In contrast, intradermal allergen exposure rarely provokes eczematous reactions, even after the late-phase response [86], underscoring the unique requirement for an epidermal route of allergen entry.

Taken together, these insights position the IgE-dependent amplification loop as an adaptive hypersensitivity module that becomes progressively assembled as type-2 inflammation intensifies and chronicity develops in extrinsic AD. Rather than representing a universal feature of all AD, it emerges as one of the most distinctive and consequential hypersensitivity-generating circuits within the broader AD spectrum.

Beyond its mechanistic implications, this framework also informs disease classification. The presence or absence of an IgE-dependent amplification loop provides a mechanistic basis for distinguishing extrinsic, indeterminate, and intrinsic forms of AD. Rather than relying solely on serum IgE levels, this framework emphasizes the functional assembly of IgE–FcεRI-mediated antigen presentation pathways—particularly the emergence of IgE-expressing LCs and IDECs—as a defining feature of the extrinsic endotype. Incorporating this mechanistic axis into AD taxonomy may help refine endotype-based stratification, improve prediction of therapeutic responses, and guide the development of precision approaches targeting IgE-dependent inflammation. However, the IgE-high and FcεRI-high endotypes discussed here are proposed as conceptual categories, not as formal diagnostic criteria.

## 5. Therapeutic Implications and Future Directions Derived from the IgE-Dependent Amplification Loop

### 5.1. IL-4/IL-13 Blockade as Modulators of the IgE-Dependent Amplification Loop

The advent of IL-4/IL-13-targeting biologics has fundamentally reshaped the therapeutic landscape of AD by interrupting the core cytokine signals that drive type-2 inflammation and IgE class switching [8]. Among these agents, dupilumab—an IL-4Rα-blocking antibody—provides the clearest window into how suppression of IL-4 and IL-13 influences the IgE-dependent amplification loop, given the central role of IL-4 in promoting IgE production and upregulating FcεRI on DCs. A growing body of clinical and immunological evidence now indicates that therapeutic responses in AD are strongly influenced by the extent to which this IgE-dependent loop remains active, and several recent studies provide converging support for this concept. Notably, these therapeutic observations are not intended as direct validation of the entire integrated model but rather as findings that align mechanistically with several components of the proposed IgE-dependent framework.

#### 5.1.1. Dupilumab-Induced Immune Remodeling

A recent long-term study [87] demonstrated that approximately three years of dupilumab therapy in adolescents and adults with AD led to significant reductions in HDM-specific IgE together with marked increases in allergen-specific IgG4, a neutralizing antibody isotype associated with immune deviation and tolerance induction. These findings suggest that prolonged IL-4/IL-13 suppression not only decreases IgE production but may also promote a shift toward a more tolerogenic humoral profile.

In contrast to these long-term humoral changes, an immunophenotyping study [88] analyzing patients after six months of dupilumab treatment demonstrated marked reductions in Th2 cells, increases in Treg frequencies, and a shift toward Th1/Th17 polarization, accompanied by significant decreases in total and HDM-specific IgE. Importantly, serum levels of soluble FcεRI and CD23 remained unchanged, indicating that although IL-4Rα blockade suppresses IgE production, it may not rapidly dismantle the structural machinery of the IgE-dependent amplification loop. This provides a mechanistic explanation for the partial responses observed in some patients treated with dupilumab [1].

#### 5.1.2. Why Dupilumab Outperforms IL-13-Only Blockade

An indirect treatment comparison between dupilumab and lebrikizumab showed that dupilumab yields a significantly higher likelihood of achieving and maintaining clinical improvement [89]. Similar findings have been reported for tralokinumab, with narrative reviews consistently demonstrating that IL-13-only blockade provides less robust and less durable efficacy compared with dupilumab in moderate-to-severe AD [90]. This pattern likely reflects the unique role of IL-4—rather than IL-13—in driving IgE class switching, FcεRI upregulation, and IgE-dependent DC activation. IL-13 blockade alone exerts only a gradual effect on suppressing IgE production and FcεRI-mediated antigen uptake, resulting in a slow and incomplete dismantling of the IgE-dependent amplification loop. Notably, these therapeutic contrasts are discussed here from a mechanistic perspective and are not intended as a systematic comparative efficacy analysis.

#### 5.1.3. Dupilumab in Combination with Adjunctive Therapies

Growing evidence suggests that combining dupilumab with therapies that directly modulate IgE-mediated pathways can yield additive or synergistic benefits in patients with extrinsic AD. A preliminary study integrating dupilumab with HDM allergen immunotherapy demonstrated greater clinical improvement than either treatment alone [91]. Dupilumab reduces IgE production and the inflammatory milieu, whereas immunotherapy promotes immune deviation and tolerance, together diminishing both the quantity of IgE and the efficiency of IgE-dependent antigen presentation.

Similarly, emerging clinical reports indicate that combining dupilumab with omalizumab provides superior outcomes in severe extrinsic AD [92,93]. Dupilumab attenuates upstream IgE generation and Th2 signaling, while omalizumab neutralizes circulating IgE and downregulates FcεRI on DCs, mast cells, and basophils. This dual targeting suppresses both the production and effector functions of IgE, offering a more comprehensive attenuation of the IgE-dependent amplification loop than monotherapy.

Moreover, the existence of intrinsic AD—where IL-4/IL-13-skewed inflammation is comparable to that of extrinsic AD despite the absence of enhanced IgE production—suggests that mechanisms beyond type-2 cytokine signaling contribute to IgE upregulation [1,13]. Considering this, the above combination therapy may be particularly rational, as it addresses both the cytokine-driven and IgE-specific components of the amplification loop.

### 5.2. Positioning the IgE-Dependent Amplification Loop in the Era of Next-Generation Targeted Therapies

Overall, the aforementioned findings suggest that:

1. IL-4Rα blockade partially suppresses but does not eliminate the IgE-dependent amplification loop.
2. Therapies that fail to reduce IgE or FcεRI signaling may show limited efficacy in IgE-high extrinsic AD.
3. Combination approaches targeting both type 2 cytokines and IgE pathways may offer superior disease control.
4. The IgE-dependent amplification loop should be recognized as a therapeutic axis distinct from type 2 cytokine signaling.

Collectively, these findings position dupilumab and related IL-4/IL-13-targeting strategies as partial but incomplete modulators of the IgE-dependent amplification loop. While they attenuate IgE production and reduce type-2 inflammation, the structural and functional components of the IgE pathway—particularly FcεRI-expressing DCs and IgE-dependent DTH—appear unlikely to improve rapidly. Recognizing these limitations helps explain the heterogeneity of clinical responses and underscores the importance of examining how other therapeutic classes engage with this amplification loop.

Beyond dupilumab and IL-13-targeting biologics, additional therapeutic classes—including IL-31 blockade, JAK inhibition, emerging upstream agents such as anti-TSLP and anti-OX40L, and omalizumab—address distinct layers of AD pathophysiology; however, none effectively or directly inhibit IgE-dependent antigen presentation. JAK inhibitors rapidly reduce pruritus and inflammation by blocking multiple cytokine pathways, yet FcεRI-expressing DCs and IgE-dependent DTH mechanisms may be insufficiently suppressed. Similarly, biologics targeting IL-31, TSLP, or OX40L alleviate itch, upstream epithelial activation, or T-cell co-stimulation, respectively, but do not dismantle the IgE-dependent loop [1,2,94].

These insights highlight the IgE-dependent amplification loop as a distinct therapeutic axis that remains insufficiently addressed by current agents. Future strategies may include:

1. Dual IL-4/IL-13–IgE pathway inhibition,
2. FcεRI-targeted therapies,
3. CD23-modulating approaches, and
4. Precision medicine frameworks identifying IgE-high or FcεRI-high endotypes.

Such approaches may be particularly relevant for patients with severe extrinsic AD, high total IgE levels, and strong environmental allergen sensitization. This recognition underscores the need to evaluate how emerging therapeutic classes interact with—or fail to influence—this amplification loop, thereby informing the development of next-generation biologics and small-molecule inhibitors.

## 6. Conclusions and Overall Synthesis

The concept of an IgE-dependent amplification loop provides a framework that integrates long-standing clinical observations with recent advances in immunology and therapeutics. While barrier dysfunction, type-2 cytokines, pruritus, and microbial dysbiosis remain central features of AD, the multilayered network formed by IgE-bearing DC-mediated antigen presentation, IgE-driven effector-cell activation, and IgE-mediated DTH contributing to spongiosis formation reveals an additional dimension of disease biology that has been largely overlooked. In this context, the framework is presented as a conceptual synthesis derived from heterogeneous lines of evidence, rather than as a fully experimentally validated circuit.

Recognizing this axis has important implications. It may help explain the heterogeneity of treatment responses across the diverse phenotypes of AD, the persistence of eczematous inflammation in IgE-high extrinsic AD, and the enhanced efficacy observed with combination approaches that simultaneously target type-2 cytokines and IgE. It also highlights the need for future therapeutic strategies aimed at directly modulating IgE-dependent antigen presentation, FcεRI-expressing DC activity, and IgE-mediated DTH. Furthermore, it offers a mechanistic axis that may refine disease classification by distinguishing IgE-dependent extrinsic AD from indeterminate and intrinsic forms. Notably, these implications should be interpreted as hypothesis-generating and reflective of the current evidence base, acknowledging that several components of the proposed loop require further empirical clarification.

Ultimately, integrating the IgE-dependent amplification loop into contemporary models of AD pathogenesis provides a more complete understanding of disease heterogeneity and establishes a rational foundation for the next generation of precision therapies. As a conceptual model, it is intended to guide future investigation and clinical stratification rather than to assert a fully resolved pathophysiological architecture.

## Data Availability

Data are contained within the article. Further inquiries can be directed to the corresponding author.

## Abbreviations

The following abbreviations are used in this manuscript:

AD	atopic dermatitis
APC	antigen-presenting cell
cDC2	conventional type 2 dendritic cell
CD	cluster of differentiation
DAPI	4′,6-diamidino-2-phenylindole
DC	dendritic cell
Der f	*Dermatophagoides farinae*
DTH	delayed-type hypersensitivity
FcεRI	high-affinity IgE receptor
HDM	house dust mite
HLA	human leukocyte antigen
IDEC	inflammatory dendritic epidermal cell
IFN	interferon
IgE	immunoglobulin E
IL	interleukin
ILC2	group 2 innate lymphoid cell
JAK	Janus kinase
LC	Langerhans cell
MHC	major histocompatibility complex
NK	natural killer (cell)
OX40L	OX40 ligand
PAR-2	protease-activated receptor-2
scRNA-seq	single-cell RNA sequencing
*S*. *aureus*	*Staphylococcus aureus*
Th	T helper
TNF	tumor necrosis factor
Treg	regulatory T cells
TSLP	thymic stromal lymphopoietin
